# Pneumococcal concentration and serotype distribution in preschool children with radiologically confirmed pneumonia compared to healthy controls prior to introduction of pneumococcal vaccination in Zanzibar: an observational study

**DOI:** 10.1186/s12879-022-07902-5

**Published:** 2022-12-10

**Authors:** Kristina Elfving, Lucia Gonzales Strömberg, Shadi Geravandi, Maria Andersson, Marc Bachelard, Mwinyi Msellem, Delér Shakely, Birger Trollfors, Rickard Nordén, Andreas Mårtensson, Anders Björkman, Magnus Lindh

**Affiliations:** 1 School of Public Health and Community Medicine, Institute of Medicine, Sahlgrenska Academy, University of Gothenburg, Göteborg, Sweden; 2 Department of Infectious Diseases, Institute of Biomedicine, Sahlgrenska Academy, University of Gothenburg, Göteborg, Sweden; 3 Department of Pediatrics, Sahlgrenska Academy, University of Gothenburg, Göteborg, Sweden; 4 Department of Clinical Microbiology, Sahlgrenska University Hospital, Göteborg, Sweden; 5 Department of Planning, Policy and Research, Ministry of Health, Zanzibar, Tanzania; 6 Department of Women’s and Children’s Health, International Maternal and Child Health (IMCH), Uppsala University, Uppsala, Sweden; 7 Malaria Research, Department of Microbiology, Tumor and Cell Biology, Karolinska Institute, Solna, Sweden

**Keywords:** Preschool child, Immunization, Serotypes, Pneumococci, Radiology, Pneumonia, Antibiotics, Sub-Saharan Africa

## Abstract

**Background:**

The World Health Organization recommends pneumococcal vaccination (PCV) in the first year of life. We investigated pneumococcal serotypes in children with clinical or radiologically confirmed pneumonia and healthy controls prior to PCV13 vaccine introduction in Zanzibar.

**Methods:**

Children (n = 677) with non-severe acute febrile illness aged 2–59 months presenting to a health centre in Zanzibar, Tanzania April–July 2011 were included. Nasopharyngeal swabs collected at enrolment were analysed by real-time PCR to detect and quantify pneumococcal serotypes in patients (n = 648) and in healthy asymptomatic community controls (n = 161). Children with clinical signs of pneumonia according to the Integrated Management of Childhood illness guidelines (“IMCI pneumonia”) were subjected to a chest-X-ray. Consolidation on chest X-ray was considered “radiological pneumonia”.

**Results:**

Pneumococcal DNA was detected in the nasopharynx of 562/809 (69%) children (70% in patients and 64% in healthy controls), with no significant difference in proportions between patients with or without presence of fever, malnutrition, IMCI pneumonia or radiological pneumonia. The mean pneumococcal concentration was similar in children with and without radiological pneumonia (Ct value 26.3 versus 27.0, respectively, p = 0.3115). At least one serotype could be determined in 423 (75%) participants positive for pneumococci of which 33% had multiple serotypes detected. A total of 23 different serotypes were identified. One serotype (19F) was more common in children with fever (86/648, 13%) than in healthy controls (12/161, 7%), (p = 0.043). Logistic regression adjusting for age and gender showed that serotype 9A/V [aOR = 10.9 (CI 2.0–60.0, p = 0.006)] and 14 [aOR = 3.9 (CI 1.4–11.0, p = 0.012)] were associated with radiological pneumonia. The serotypes included in the PCV13 vaccine were found in 376 (89%) of the 423 serotype positive participants.

**Conclusion:**

The PCV13 vaccine introduced in 2012 targets a great majority of the identified serotypes. Infections with multiple serotypes are common. PCR-determined concentrations of pneumococci in nasopharynx were not associated with radiologically confirmed pneumonia.

*Trial registration* Clinicaltrials.gov (NCT01094431).

## Introduction

Infections with *Streptococcus pneumoniae* are among the most important causes of illness and death in preschool children, of which half occur in Africa [1]. Otitis media is the most common clinical manifestation, whereas most deaths are caused by a septicaemia associated with meningitis or pneumonia [2].

*Streptococcus pneumoniae* is usually transmitted through respiratory droplets from preschool children that constitute the main reservoir. Pneumococcal colonisation of the nasopharynx begins during the first month of life [3–5], and is a prerequisite for symptomatic infections.

More than 100 pneumococcal serotypes have been described, of which some are known to cause more serious invasive disease [6]. In 2007, the World Health Organization (WHO) recommended global implementation of a seven-valent conjugate pneumococcal vaccine targeting the most prevalent and severe serotypes in the childhood immunization programmes, in particular in countries with a high child mortality [7]. More than 80% of African countries have introduced pneumococcal vaccination (PCV) into their child immunization programme following the 2012 updated WHO policy that recommended implementation of a 10- or 13-valent PCV [8]. In 2019, the estimated coverage of a third PCV dose was 70% in Africa (https://www.who.int/data/gho/data/indicators/indicator-details/GHO/pneumoccocal-conjugate-vaccines-(pcv3)-immunization-coverage-among-1-year-olds-). Despite implementation of vaccination, Africa still has the heaviest burden of pneumococcal disease and the highest death toll [1, 9]. Vaccine effectiveness studies in both high and low income countries have shown a significant decrease of the prevalence of the most common serotypes. However, the effect of vaccination on the frequencies and serotypes of pneumococci in nasopharynx is uncertain, since studies conducted in Tanzania before the introduction of PCV in 2012 are few and small [10].

It is well established that the prevalence of pneumococcal infections in the nasopharynx in children is higher in low resource settings in comparison with high-income countries [11]. Due to relatively few cases and lacking data for invasive pneumococcal disease (IPD), in particular from Africa, the detection and bacterial density of pneumococci in the nasopharynx may be used as proxy indicators of radiological pneumonia and IPD [12–14], particularly when studying vaccine efficacy and serotype replacement [8, 15–18].

Real-time PCR for both detection and typing has higher sensitivity and specificity compared to traditional methods and appears better to identify multiple serotypes in one and the same patient [19, 20].

The aim of this study was to describe and compare which pneumococcal serotypes that infect the nasopharynx of healthy controls and febrile children with and without radiologically confirmed pneumonia prior to the introduction of pneumococcal vaccination in Zanzibar.

## Methods

### Study design, enrolment and follow-up of patients

This was a prospective short-term longitudinal observational cohort study that was part of a larger project with the primary aim to investigate causes of acute uncomplicated febrile illness in children presenting to Kivunge primary health care centre in Zanzibar, Tanzania between April and July 2011 (results published: [21]). Inclusion criteria were: children 2–59 months of age; history of fever in the preceding 24 h or confirmed fever (axillary temperature of ≥ 37.5 °C by electronic thermometer); and written informed proxy consent from a legal guardian. Exclusion criteria were: severe disease according to Integrated Management of Childhood Illness guidelines (IMCI) [21] previous study inclusion; and reported inability to come for follow-up visits.

On inclusion day, all included study patients had a nasopharyngeal swab sampled, in addition to the clinical information collected on a study specific case record form. Follow up visits were conducted on day four (± 2 days) and 14 (± 2 days) and caretakers were instructed to return with their children in case any signs of severe disease occurred. A child that did not return for a scheduled follow-up, was actively traced in their home. Any patient with signs of severe illness (IMCI definition), predefined abnormal laboratory values or withdrawal of consent during the 14-day follow-up was discontinued.

### Radiology

Children with signs of pneumonia according to IMCI, i.e. rapid breathing and cough and/or breathing difficulties (“IMCI pneumonia”) were subjected to a chest-X-ray (CXR). Analog CXRs were performed on-site using an anterior–posterior view according to WHO recommended standards and were within three days interpreted by a radiologist in the main referral hospital on the island (Mnazi Mmoja hospital) as either: primary endpoint consolidation, other infiltrate, normal, or un-interpretable, i.e. not of sufficient quality [22]. End-point consolidation was considered “radiological pneumonia”. In case of any significant severe pathology observed on the CXR that required immediate referral to the hospital for intervention, the patient was discontinued from the study and referred to a designated paediatric specialist in Zanzibar main referral hospital. After field study completion, all CXR-films were digitalised according to WHO standards [22] and interpreted by an experienced paediatric radiologist in Sweden. All discordant CXR results regarding “primary endpoint consolidation” or “no primary endpoint consolidation” between the two readers were subjected a third and decisive interpretation by an experienced radiologist in Sweden blinded to the previous radiologists’ reports.

In Tanzania and Zanzibar, introduction of pneumococcal vaccination with PCV13 started in early 2012, just after completion of the present study.

### Enrolment of healthy controls

Healthy controls were recruited during the same study time period from eight villages in the catchment area. Each study week, one of the villages was visited and asymptomatic children were identified through house-to-house screening. Inclusion criteria for eligible healthy controls were: age 2–59 months with no history of cough, runny nose or fever (by history and/or electronic axillary temperature < 37.5 °C) in the preceding ten days; no previous participation in the study; and a written informed proxy consent from a caretaker. Recruitment was unmatched but aimed at a balanced age, sex and geography-distribution. Eligible children (maximum one child per household) provided nasopharyngeal swabs for PCR-analyses. Anthropometric data was collected only in patients.

### Sampling

The swabs (Copan Regular Flocked Swab 502CS01, Copan Italia Spa, Brescia, Italy) were sampled according to study specific standard operating procedures (SOPs). The swab was inserted into the far back of the nasopharynx of the child which was seated in the lap of the accompanying care taker. The swab was rotated for a couple of seconds, and then pulled out quickly. Directly after sampling, the swab was placed into a sterile tube containing 1 mL sterile NaCl 0.9%. Within 2 h after sampling the swabs and the saline were vortexed. The liquid content was transferred to a micro-tube using a disposable transfer pipette and stored in controlled temperature of − 70 °C. After field trial completion, all samples were transported to Sweden on dry ice for molecular analyses.

### Nucleic acid extraction and real-time polymerase chain reaction

Nasopharyngeal swab solution (180 µL) was mixed with 20 µL PBS 10 ×. This volume was used for extraction of total nucleic acid using the MagNA Pure LC instrument (Roche Diagnostics, Mannheim, Germany) and the Total Nucleic Acid Kit (Roche Diagnostics) according to manufacturer’s instruction. The nucleic acids were eluted in 100 µL and of these, 5 µL was used for each polymerase chain reaction (PCR) assay.

### Detection of *S. pneumoniae*

Initially, *S. pneumoniae* was identified by real-time PCR targeting the spn9828 gene [23], using the forward primer SpneumF TTTCTGGATAGAGGGAGTATCCGA (300 nM), reverse primer SpneumR2 TTACCAACCTACTCATCTTCTCACCA (300 nM), and probe SpneumP_MGB CAAAGTTAATACCGCCCTC (200 nM) in a 20 µL real-time PCR reaction with Universal Mastermix (Applied Biosystems). After an initial step at 50 °C for 2 min followed by 10 min of denaturation at 95 °C, 45 cycles of two-step PCR was preformed (15 s at 95 °C, 60 s at 58 °C) in a Quant Studio 6 Flex (Applied Biosystems, Foster City, CA). The cpsA gene target that was included in the serotyping panel, was an additional support for pneumococcus identification. Furthermore, real-time PCR that targets the ‘Xisco gene’ [24], which has been shown to be even more specific for *S. pneumoniae,* was applied on a subset of the samples. If a sample was positive for the spn9828 gene but negative in the cpsA gene PCR or in subtyping, the classification as non-pneumococcus spp or pneumococcus spp was based on the ‘Xisco gene’ analysis. If the sample was negative for the spn9828 gene but positive in the cpsA gene PCR and in subtyping, the sample was regarded as a pneumococcus. For all targets, a Ct cut-off value of 40 was applied. The third step “Xisco” analysis was conducted only on those samples with a Ct value < 35 in the spn9828 gene analysis.

### Serotype identification

Serotype identification was done using a multiplex PCR panel that is able to identify 40 serotypes (some in combinations), performed according to Gonzales-Siles, et al. [25]. The result for each serotype was recorded as the threshold cycle (Ct) value, which is inversely related to the pathogen load for each target. A Ct cut-off value of 40 was applied.

### Ethical considerations

The study was conducted in accordance with the Declaration of Helsinki and Good Clinical Practice and registered at Clinicaltrials.gov (NCT01094431, 29/03/2010). The study was approved by the Zanzibar Medical Research Ethics Committee in Tanzania (Reference number: ZAMREC/0001/April/010), and by the Regional Ethical Review Board in Gothenburg, Sweden (Reference number: 266-10). A written and informed proxy consent was taken from the guardian of all study participants before inclusion. No financial incentives were given.

### Data management and statistical analysis

This was an exploratory study, without need for sample size calculation. Prior to the parent study [21] it was estimated that inclusion of 650 patients and 150 controls would be sufficient. Data was double-entered in CSPro or Excel, validated and exported to STATA® 14 (StataCorp). 2015. *Stata Statistical Software: Release 14*. College Station, TX: StataCorp LP.) where all statistical analyses were performed. p-values < 0.05 were considered statistically significant. Fisher’s exact test and exact binomial test was used for binary data and proportions, and two-sample t-test for comparisons of means and Mann–Whitney U test for comparison of medians. Logistic regression was performed to assess the association between the independent variables age, sex, and all the pneumococcal serotype variables (binary variable; positive/negative) and the outcomes/dependent variables radiological pneumonia, and IMCI pneumonia, respectively. Those serotype variables that had a p-value < 0.25 when comparing proportions of detection in the respective groups (Fisher’s test) were included in a multivariable logistic regression model that also adjusted for age (months) and sex of the patient.

## Results

A total of 844 participants were included of whom 809 (648 patients and 161 healthy controls) were sampled for pneumococcal analysis. Demographic characteristics of patients and healthy controls were similar except for age which was higher among healthy controls (median age 24 months) than in patients (median age 14 months) (Table 1). Overall, 69% (562/809) had a pneumococcal infection. Similar proportions were observed in patients (459/648; 70%) and in healthy controls (103/161; 64%; p = 0.104). Of the 809 patients tested for pneumococci, 294 of the 369 that fulfilled the IMCI criteria for pneumonia underwent a CXR, which showed radiological pneumonia in 13% (39/294). Two children died, and in one of them *S. pneumoniae* (serogroup 6) was detected in nasopharynx.

**Table 1:** Demographic, socioeconomic and clinical characteristics of study participants

Characteristics	Patients	Healthy controls
Number of enrolled participants	677 (100%)	167 (100%)
Female	325 (48%)	81 (49%)
Age		
Median age months (IQR)	14 (9–24)	24 (12–36)
2–11 months	232 (34%)	32 (19%)
12–23 months	231 (34%)	42 (25%)
24–35 months	109 (16%)	41 (25%)
36–59 months	105 (16%)	52 (31%)
Care taker level of education		
No school education	253 (37%)	
> 6 years education	317 (47%)	
Breastfeeding children (a) < 24 months	434 (94%)^a^	
Fully immunized^b^ > 11 months	420 (94%)^c^	
Antibiotics consumed before study inclusion	56 (8%)	
Underweight; % below -2SD (CI)	28.3 (24.9–31.8)	
Temperature		
Median temperature (IQR)	37.3 (36.8–38.0)	
36.0–37.4	375 (55%)	
37.5–39.0	240 (35%)	
> 39.0	46 (7%)	
Severe clinical picture after study inclusion	29 (4%)	
Most common main complaints		
Fever	644 (95%)	
Cough	580 (86%)	
Runny nose	455 (67%)	
Vomiting	34 (5%)	
Ear pain	21 (3%)	
Antibiotic prescription on day 0	500 (74%)	
One type of antibiotic	394 (79%)	
Two types of antibiotics	103 (21%)	
Beta lactam antibiotics/parenteral benzyl penicillin	470 (93%) / 112 (17%)	
Trimethoprim/sulfamethoxazole	74 (11%)	
Other types of antibiotics	95 (14%)	

n ≤ 10 missing if not indicated)

*IQR* Interquartile range

^a^Denominator for patients < 24 months: n = 463

^b^BCG, OPV3, Pentavalent/DPT3, measles

^c^Denominator for patients > 11 months: n = 445

As shown in Table 2, detection of pneumococci in the nasopharynx was not associated with measured fever, malnutrition, IMCI pneumonia, radiological pneumonia, or development of severe disease after inclusion.

**Table 2:** Clinical characteristics of patients with or without *S. pneumoniae* detected in their nasopharynx

	*S. pneumonia* n (%) if not stated otherwise			
	All (n = 648^a^)	Positive (n = 459^a^)	Negative (n = 189^a^)	p-value^b^
IMCI pneumonia	369/585 (63%)	262 (62%)	107 (67%)	0.338
Radiological pneumonia	39/331 (12%)	31 (13%)	8 (8%)	0.263
Febrile	255/648 (41%)	180 (39%)	75 (40%)	0.9
Prior antibiotic consumption	53/648 (8%)	31 (7%)	22 (12%)	0.057
Moderate malnutrition^c^	180/647 (28%)	125 (27%)	55 (29%)	0.631
Severe malnutrition^d^	48/647 (7%)	29 (6%)	19 (10%)	0.136
Mean CRP (mg/dL)	25.6	27.0	22.1	0.07^e^
Median CRP (mg/dL)	13	14	12	0.04^f^
Severe outcome	27/648 (4%)	17 (3.7%)	10 (5.3%)	0.389
Mean age (months)	19.2	18.7	20.5	0.11
Female	314/648 (48%)	220 (48%)	94 (50%)	0.730

*CRP* C-reactive protein, *IMCI* Integrated Management of Childhood illnesses guidelines

^a^All tested for *S. pneumoniae*

^b^Fisher's exact test, two-sided

^c^(W/A < − 2zScore)

^d^(W/A < − 3zScore)

^e^t-test

^f^Mann Whitney U test

Pneumococcus negative patients had a tendency for higher frequency of prior antibiotic consumption than positive patients (7% versus 12%, p = 0.057) (Table 2). If disregarding those patients that had received antibiotics before, the mean Ct value (proxy for inverse concentration of pneumococcal DNA) was similar in patients with or without radiological pneumonia (Ct 26.3 vs. 27.0, p = 0.3115) as well as when comparing patients and healthy controls (Ct 27.9 versus 27.5, p = 0.3005). The mean bacterial concentration was approximately ten times higher in serotyping-positive as compared with serotyping-negative cases (26.4 (CI 26.1–26.7) vs. Ct 30.0 (CI 29.5–30.5), p < 0.00001.

### Pneumococcal serotypes and clinical picture

Of all 562 participants positive for pneumococci, one or several pneumococcal serotypes were detected in 423 (75%), of which 141 (33%) participants had multiple serotypes detected. In 139 (17%) it was not possible to identify the serotype, implying that those patients likely carried serotypes that are not included in the serotyping panel. In total 173 (31%) of 562 participants sampled were positive for serogroup 6.

There was a similar distribution of pneumococcal serotypes in patients and healthy controls except for serotype 19F which was more common in patients (13% vs. 7%, p = 0.043) (Table 3). Yet, serotype 19F was also more common in patients without signs of IMCI pneumonia (18% vs 12%, p = 0.048) (Table 4) whereas patients with radiological pneumonia had significantly higher detection of serotypes 9A/V than patients with no signs of pneumonia on CXR (8% vs 1%, p = 0.024). (Table 4). By multivariable regression including the variables age, gender and all analysed pneumococcal serotypes (positive/negative) with a p-value < 0.25 (see Table 4), serotype 9A/V (aOR = 10.9 (CI 2.0–60.0, p = 0.006) and also serotype 14 (aOR = 3.9 (CI 1.4–11.0, p = 0.012) were significantly associated with radiological pneumonia whereas serotype 19F was negatively associated with IMCI pneumonia (Table 5).

**Table 3:** Real time PCR detection of *S. pneumoniae* serotypes in patients and healthy controls

Serogroup/type, order of frequency	All participants^ab^	Patients^b^	Healthy controls	p-value^c^
	(n = 809)	(n = 648)	(n = 161)	Patients vs controls
6A/B/C/D	143 (18%)	115 (18%)	28 (17%)	1.000
19F	98 (12%)	86 (13%)	12 (7%)	0.043
19A	45 (6%)	38 (6%)	7 (4%)	0.566
14	44 (5%)	39 (6%)	5 (3%)	0.175
23F	31 (4%)	25 (4%)	6 (4%)	1.000
15BC	30 (4%)	24 (4%)	6 (4%)	1.000
11	29 (4%)	25 (4%)	4 (2%)	0.486
7C	15 (2%)	14 (2%)	1 (1%)	0.327
5	12 (1%)	11 (2%)	1 (1%)	0.477
10A	12 (1%)	10 (2%)	2 (1%)	1.000
9A/V	9 (1%)	8 (1%)	1 (1%)	1.000
18	7 (1%)	5 (1%)	2 (1%)	0.631
12	7 (1%)	7 (1%)	0 (0%)	0.356
3	7 (1%)	6 (1%)	1 (1%)	1.000
20	5 (1%)	4 (1%)	1 (1%)	1.000
4	4 (0%)	3 (0%)	1 (1%)	1.000
Negative in serotyping	386 (48%)	296 (46%)	90 (56%)	0.022
One serotype detected	283 (35%)	233 (36%)	50 (31%)	0.268
Two serotypes detected	111 (14%)	93 (14%)	18 (6%)	0.370
> 3 serotypes detected	29 (4%)	26 (4%)	3 (2%)	0.240

^a^For pneumococcal serotypes n < 3 and are not displayed here: *S. pneumoniae* spp positive: 38, 22FA, 17, 1 (n = 2), 2, 8, 9NL (n = 1) 33, 7FA (n = 0)

^b^All tested for *S. pneumoniae*

^c^Fisher’s exact test, two-sided

Serotypes included in PCV7: 4, 6B, 9 V, 14, 18C, 19F, 23F. Serotypes included in PCV10: 1, 4, 5, 6B, 7F, 9V, 14, 18C, 19F, 23F Serotypes included in PCV13: 1, 3, 4, 5, 6A, 6B, 7F, 9V, 14, 18C, 19A, 19F, 23F

**Table 4:** Detection of *S. pneumoniae* serotypes by real time PCR detection of in patients with or without radiological pneumonia and WHO/IMCI pneumonia

Serogroup/type, order of frequency	Patients with IMCI pneumonia^a^	Patients without IMCI pneumonia^a^	p-value^b^	Patients with radiological pneumonia	Patients without radiological pneumonia	p-value^b^
	(n = 369)	(n = 216)		(n = 39)	(n = 292)	
6A/B/C/D	68 (18%)	42 (19%)	0.827	6 (15%)	54 (18%)	0.825
19F	43 (12%)	38 (18%)	0.048	1 (3%)	37 (13%)	0.064
19A	21 (6%)	14 (6%)	0.720	3 (8%)	16 (5%)	0.478
14	28 (8%)	8 (4%)	0.074	6 (15%)	19 (7%)	0.097
23F	13 (4%)	11 (5%)	0.391	3 (8%)	9 (3%)	0.157
15BC	11 (3%)	11 (5%)	0.259	1 (3%)	10 (3%)	1.000
11	15 (4%)	8 (4%)	1.000	3 (8%)	9 (3%)	0.157
7C	9 (2%)	2 (1%)	0.344	0 (0%)	7 (2%)	1.000
5	6 (2%)	3 (1%)	1.000	0 (0%)	5 (2%)	1.000
10A	9 (2%)	1 (1%)	0.101	1 (3%)	7 (2%)	1.000
9A/V	6 (2%)	2 (1%)	0.717	3 (8%)	3 (1%)	0.024
18	2 (1%)	3 (1%)	0.364	0 (0%)	2 (1%)	1.000
12	3 (1%)	3 (1%)	0.675	0 (0%)	2 (1%)	1.000
3	3 (1%)	3 (1%)	0.675	0 (0%)	2 (1%)	1.000
4	2 (1%)	1 (0%)	1.000	1 (3%)	0 (0%)	0.118

Serotypes positive in patients checked for radiological pneumonia: (Pneumococcus 20 (n = 4), 17 (n = 2), 22FA, 8, 4 (n = 1) 38, 33, 9 NL, 7FA, 2, 1 (n = 0)

Serotypes positive in patients checked for IMCI pneumonia: Pneumococcus 20, (n = 4), 38, 22FA, 17, 1 (n = 2), 4 (n = 3), 9NL, 8, 2 (n = 1), 33, 7FA (n = 0)

^a^All tested for *S. pneumoniae*

^b^Fisher’s exact test, two-sided

**Table 5:** Multiple logistic regression analysis of pneumococcal serotypes associated with IMCI/WHO pneumonia and radiological pneumonia

	IMCI pneumonia		Radiological pneumonia	
	Adjusted OR (CI)	p	Adjusted OR (CI)	p
Age	0.96 (0.95–0.98)	< 0.001	1.03 (1.00–1.06)	0.031
Gender	0.88 (0.6–1.3)	0.484	0.66 (0.32–1.33)	0.246
Serotypes				
14	1.7 (0.7–3.8)	0.22	3.9 (1.4–11.1)	0.012
19F	0.6 (0.3–0.9)	0.024	0.2 (0.03–1.8)	0.16
9A/V	0.3–9.0	0.528	10.9 (2.0–60.0)	0.006
11	–^a^	–^a^	3.5 (0.9–14.3)	0.081
23F	–^a^	–^a^	3.4 (0.8–13.8)	0.091
10A	5.1 (0.6–41.5)	0.124	–^a^	–^a^

^a^p ≥ 0.25 in univariate analysis (Table 4)

### Vaccine serotypes and clinical picture

**Fig. 1:**
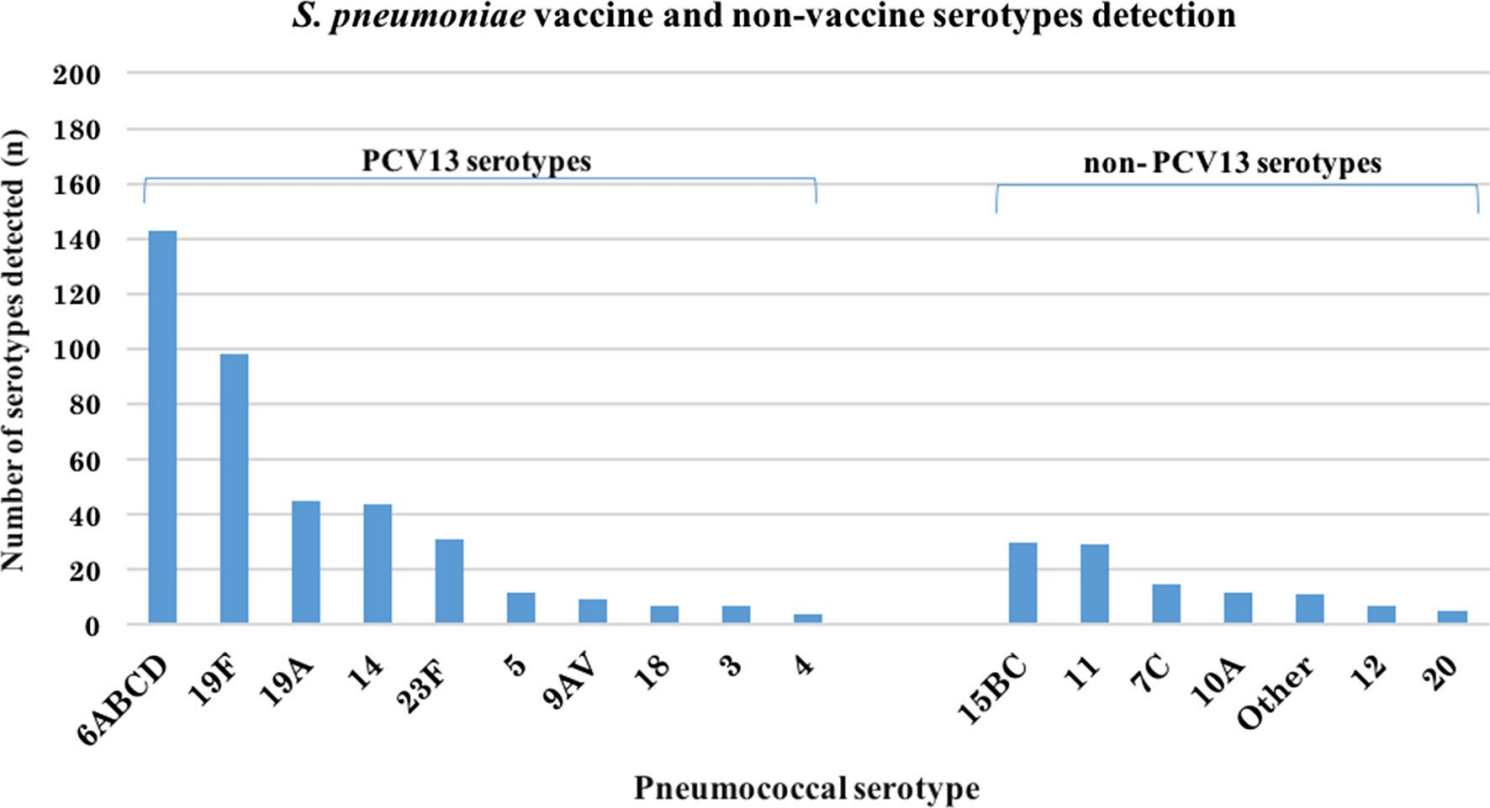
Pneumococcal serotypes grouped according to PCV13 vaccine types and non-PCV 13 vaccine types

A PCV13 serotype was found in 376 (46%) of all 809 participants. The most common PCV13 vaccines serotypes were 6ABCD, 19F, 19A, 14, and 23F whereas 15BC, 11, 7C, 10A were the most common non-vaccine serotypes (see Fig. 1). There was neither any difference in the prevalence of radiological pneumonia nor of IMCI pneumonia when comparing patients infected with PCV13 and non-PVC13 vaccine serotypes, 23/160 (14.3%), 16/171 (9.4%), (p = 0.175) and 177/293 (60%), (192/292 (66%) (p = 0.199), respectively (Table 4).

## Discussion

This study on distribution and clinical picture of patients infected with pneumococcal serotypes was conducted before PCV13 vaccine introduction in Zanzibar. The spectrum of pneumococcal serotypes among febrile children was investigated, as well as the association of pneumococcal detection and density with clinical signs and symptoms. We found a high rate of pneumococcal infection in nasopharynx and the observed serotypes were similar to those detected in previous pneumococcal epidemiological studies [20, 26–37]. The clinical picture of the study participants did not depend on the serotypes with which they were infected, with some few exceptions.

Overall, serogroup/types 6, 14,19A, and 19F were the most commonly detected in the nasopharynx of the children. The results correspond well with previous reports on serotypes found in children with invasive pneumococcal disease prior to PCV introduction both globally [3, 38] and in Africa [28, 35–37], as well as similar studies performed in Africa including East Africa [20, 26–34]. Almost half of the patients carried a PCV13 vaccine serotype in their nasopharynx similar to previous studies conducted before or early after PCV13 introduction (ranging from 49.7–68%) [26, 27, 33, 34, 39]. The addition of PCR analyses to culture negative specimens increase both overall detection of pneumococci [40], and the crude detection of serotypes [40], as well as detection of multiple serotypes in one patient, in one study from 5 to 30% [20]. The latter is in line with our detection of multiple serotypes in 33%. Sequence-based methods have the advantage of detecting all serotypes described but in a qualitative manner compared to real-time PCR which gives quantitative results based on Ct values.

The clinical spectrum of pneumococcal infection from asymptomatic nasopharyngeal infection, to otitis media, pneumonia or septicaemia is ruled by a complex interplay between the host, its environment and the pathogen. Still, most important for outcome of invasive pneumococcal disease is the pneumococcal serotype/capsule type with which the patient is infected [41]. Indeed, in a meta-analysis on IPD by Weinberger et al., in patients with bacteraemic pneumonia, serotypes 1, 7F, and 8 were associated with decreased relative risks, and serotypes 3, 6A, 6B, 9N, and 19F with increased relative risks [42] of death. In our study serotypes (9A/V and 14) were more common in patients with radiological pneumonia than without. Both are included in the PCV13 vaccine that was introduced in 2012 in Zanzibar. To our knowledge few studies [43], and none in Africa, conducted before the PCV introduction have reported serotype specific prevalence in children with chest X-ray verified pneumonia, and compared with simultaneously recruited control subjects. Another strength of the present study is that the method used to decide on the outcome “radiological pneumonia” has also been used in pneumococcal vaccine effectiveness studies. The method is in a recent re-evaluation report still regarded a good proxy indicator for bacterial (pneumococcal) pneumonia [44]. Indeed, studies have shown a greater reduction of CXR-verified pneumonia after PCV introduction in comparison with the level of reduction of IMCI pneumonia (diagnosis made on merely clinical parameters) [45], which has recently also been shown in Africa [46].

Coherently, there was no difference in pneumococcal serotypes detected in the nasopharynx of IMCI pneumonia patients in comparison with non-IMCI pneumonia patients, as also reported by others [40, 47–49]. One exception is the serotype 19F which was more common in patients without pneumonia symptoms (rapid breathing), and more common in patients than healthy controls overall. Serotype 19F has been reported as a common pathogen in nasopharyngeal samples [10], but not so prone to invasiveness in patients without co-morbidities [50]. Also, 19F colonisation was not more common in patients with radiological pneumonia than without in a study from Mozambique conducted after PCV introduction [40]. Yet, Birindwa et al. showed that a third of patients with a fatal outcome carried 19F in their nasopharynx [39]. Only 2 of our study participants were infected with serotype 1, which is seldom found as a colonizer of the nasopharynx but has been regarded a pathogen of concern due to its high relative risk to cause invasive [37] and complicated pneumococcal disease like empyema [51] and meningitis [52].

The proportion of pneumococcal infection in the upper respiratory tract was higher in the present study (70%) that used real-time PCR detection from oro/nasopharyngeal swabs than in previous African studies that use culture as detection method (20–60% prevalence) [11, 15, 27, 53–55]. Yet, a few studies, some of which conducted additional PCR on culture negative samples, have reported rates as high as 80–90% [26, 40]. The difference in pneumococcal detection between studies is likely often a matter of methodology [11, 56] which makes comparison between studies difficult.

The present study observed a similar pneumococcal concentration in children with and without radiologically verified pneumonia. This agrees with some previous studies [48], but contrasts to other studies that have shown an association between higher pneumococcal quantity and more severe disease. One reported an increased rate of invasive infection in patients with high pneumococcal bacterial load [12], and another that pneumococcal density was higher in pneumonia than in non-pneumonia patients [13]. PCR Ct-value cut offs have accordingly been proposed to identify pneumonia [14], but this is not supported by our results.

A low pneumococcal concentration was the probable reason that a sixth of the PCR positive samples could not be serotyped (defined as high pneumococcal Ct value) since the Ct values were significantly higher in serotyping negative patients. Another reason could be that the sample contained a serotype that was not targeted by the serotyping PCR, or a so called non-typeable pneumococcus (NTP). Yet, NTPs have rarely or never been reported to cause invasive pneumococcal disease in Africa [35] or USA [57], with less than 0.1% of the IPD-strains being non-typeable pneumococci. The pneumococcal serotypes that were analysed in this study were very similar to previous studies with the exception of serotype 16 which was not included in our panel, since it has rarely been identified as an IPD serotype. Others have found a significant proportion of the patients infected with serotype 16 (> 10%) [26, 31].

It is interesting that a non-vaccine serotype was found in slightly more than half of the cases in this study, conducted before the introduction of pneumococcal vaccine. In other areas of the world the non-vaccine serotypes have emerged after introduction of pneumococcal conjugate vaccines [58–60]. With increasing likelihood of serotype replacement, more updated detection methods and serotype panels are required.

## Conclusion

The study conducted in 2011, prior to the introduction of the pneumococcal immunization in Zanzibar in 2012, showed that one third of children were infected with multiple pneumococcal serotypes. Pneumococcal concentration in nasopharynx was not associated radiological pneumonia. A few serotypes were more common in symptomatic children than healthy controls, and all of them were included in the PCV13 vaccine. A pneumococcus serotype corresponding to those included in the PCV13 vaccine was detected in the nasopharynx of almost half of all study participants.

## Data Availability

The datasets used and/or analysed during the current study are available from the corresponding author on reasonable request.

## Abbreviations

Ct: Threshold cycle
CXR: Chest-X-ray
IMCI: Integrated Management of Childhood Illness guidelines (IMCI)
IPD: Invasive pneumococcal disease
PCV: Pneumococcal vaccination
WHO: The World Health Organization

## References

[1] Wahl B, O'Brien KL, Greenbaum A, Majumder A, Liu L, Chu Y (2018). Burden of *Streptococcus pneumoniae* and *Haemophilus influenzae* type b disease in children in the era of conjugate vaccines: global, regional, and national estimates for 2000–15. Lancet Glob Health.

[2] Collaborators GBDLRI (2018). Estimates of the global, regional, and national morbidity, mortality, and aetiologies of lower respiratory infections in 195 countries, 1990–2016: a systematic analysis for the Global Burden of Disease Study 2016. Lancet Infect Dis.

[3] Bogaert D, De Groot R, Hermans PW (2004). *Streptococcus pneumoniae* colonisation: the key to pneumococcal disease. Lancet Infect Dis.

[4] Heinsbroek E, Tafatatha T, Phiri A, Swarthout TD, Alaerts M, Crampin AC (2018). Pneumococcal carriage in households in Karonga District, Malawi, before and after introduction of 13-valent pneumococcal conjugate vaccination. Vaccine.

[5] Heinsbroek E, Tafatatha T, Chisambo C, Phiri A, Mwiba O, Ngwira B (2016). Pneumococcal acquisition among infants exposed to HIV in rural Malawi: a longitudinal household study. Am J Epidemiol.

[6] Geno KA, Gilbert GL, Song JY, Skovsted IC, Klugman KP, Jones C (2015). Pneumococcal capsules and their types: past, present, and future. Clin Microbiol Rev.

[7] WHO (2007). Pneumococcal conjugate vaccine for childhood immunization—WHO position paper. Wkly Epidemiol Rec.

[8] Deloria Knoll M, Bennett JC, Garcia Quesada M, Kagucia EW, Peterson ME, Feikin DR (2021). Global landscape review of serotype-specific invasive pneumococcal disease surveillance among countries using PCV10/13: the pneumococcal serotype replacement and distribution estimation (PSERENADE) project. Microorganisms.

[9] O'Brien KL, Wolfson LJ, Watt JP, Henkle E, Deloria-Knoll M, McCall N (2009). Burden of disease caused by *Streptococcus pneumoniae* in children younger than 5 years: global estimates. Lancet.

[10] Moyo SJ, Steinbakk M, Aboud S, Mkopi N, Kasubi M, Blomberg B (2012). Penicillin resistance and serotype distribution of *Streptococcus pneumoniae* in nasopharyngeal carrier children under 5 years of age in Dar es Salaam, Tanzania. J Med Microbiol.

[11] Adegbola RA, DeAntonio R, Hill PC, Roca A, Usuf E, Hoet B (2014). Carriage of *Streptococcus pneumoniae* and other respiratory bacterial pathogens in low and lower-middle income countries: a systematic review and meta-analysis. PLoS ONE.

[12] Wolter N, Tempia S, Cohen C, Madhi SA, Venter M, Moyes J (2014). High nasopharyngeal pneumococcal density, increased by viral coinfection, is associated with invasive pneumococcal pneumonia. J Infect Dis.

[13] Baggett HC, Watson NL, Deloria M, Brooks WA, Feikin DR, Hammitt LL (2017). Density of upper respiratory colonization with *Streptococcus pneumoniae* and its role in the diagnosis of pneumococcal pneumonia among children aged <5 years in the PERCH Study. Clin Infect Dis.

[14] Bjarnason A, Lindh M, Westin J, Andersson LM, Baldursson O, Kristinsson KG (2017). Utility of oropharyngeal real-time PCR for *S. pneumoniae* and *H. influenzae* for diagnosis of pneumonia in adults. Eur J Clin Microbiol Infect Dis.

[15] Usuf E, Bottomley C, Adegbola RA, Hall A (2014). Pneumococcal carriage in sub-Saharan Africa–a systematic review. PLoS ONE.

[16] van Gils EJ, Veenhoven RH, Hak E, Rodenburg GD, Bogaert D, Ijzerman EP (2009). Effect of reduced-dose schedules with 7-valent pneumococcal conjugate vaccine on nasopharyngeal pneumococcal carriage in children: a randomized controlled trial. JAMA.

[17] Lindstrand A, Galanis I, Darenberg J, Morfeldt E, Naucler P, Blennow M (2016). Unaltered pneumococcal carriage prevalence due to expansion of non-vaccine types of low invasive potential 8 years after vaccine introduction in Stockholm, Sweden. Vaccine.

[18] Nzenze SA, Madhi SA, Shiri T, Klugman KP, de Gouveia L, Moore DP (2017). Imputing the direct and indirect effectiveness of childhood pneumococcal conjugate vaccine against invasive pneumococcal disease by surveying temporal changes in nasopharyngeal pneumococcal colonization. Am J Epidemiol.

[19] Satzke C, Dunne EM, Porter BD, Klugman KP, Mulholland EK, PneuCarriage project g (2015). The PneuCarriage Project: a multi-centre comparative study to identify the best serotyping methods for examining pneumococcal carriage in vaccine evaluation studies. PLoS Med.

[20] Olwagen CP, Adrian PV, Madhi SA (2017). Comparison of traditional culture and molecular qPCR for detection of simultaneous carriage of multiple pneumococcal serotypes in African children. Sci Rep.

[21] Elfving K, Shakely D, Andersson M, Baltzell K, Ali AS, Bachelard M (2016). Acute uncomplicated febrile illness in children aged 2–59 months in Zanzibar-aetiologies, antibiotic treatment and outcome. PLoS ONE.

[22] Cherian T, Mulholland EK, Carlin JB, Ostensen H, Amin R, de Campo M (2005). Standardized interpretation of paediatric chest radiographs for the diagnosis of pneumonia in epidemiological studies. Bull World Health Organ.

[23] Suzuki N, Yuyama M, Maeda S, Ogawa H, Mashiko K, Kiyoura Y (2006). Genotypic identification of presumptive *Streptococcus pneumoniae* by PCR using four genes highly specific for *S. pneumoniae*. J Med Microbiol.

[24] Salva-Serra F, Connolly G, Moore ERB, Gonzales-Siles L (2018). Detection of "Xisco" gene for identification of *Streptococcus pneumoniae* isolates. Diagn Microbiol Infect Dis.

[25] Gonzales-Siles L, Salva-Serra F, Degerman A, Norden R, Lindh M, Skovbjerg S (2019). Identification and capsular serotype sequetyping of *Streptococcus pneumoniae* strains. J Med Microbiol.

[26] Kobayashi M, Conklin LM, Bigogo G, Jagero G, Hampton L, Fleming-Dutra KE (2017). Pneumococcal carriage and antibiotic susceptibility patterns from two cross-sectional colonization surveys among children aged < 5 years prior to the introduction of 10-valent pneumococcal conjugate vaccine-Kenya, 2009–2010. BMC Infect Dis.

[27] Jroundi I, Mahraoui C, Benmessaoud R, Moraleda C, Munoz Almagro C, Seffar M (2017). *Streptococcus pneumoniae* carriage among healthy and sick pediatric patients before the generalized implementation of the 13-valent pneumococcal vaccine in Morocco from 2010 to 2011. J Infect Public Health.

[28] Sigauque B, Verani JR, Massora S, Vubil D, Quinto L, Acacio S (2018). Burden of invasive pneumococcal disease among children in rural Mozambique: 2001–2012. PLoS ONE.

[29] Verani JR, Massora S, Acacio S, Dos Santos RT, Vubil D, Pimenta F (2018). Nasopharyngeal carriage of *Streptococcus pneumoniae* among HIV-infected and -uninfected children <5 years of age before introduction of pneumococcal conjugate vaccine in Mozambique. PLoS ONE.

[30] Donkor ES, Annan JA, Badoe EV, Dayie NT, Labi AK, Slotved HC (2017). Pneumococcal carriage among HIV infected children in Accra, Ghana. BMC Infect Dis.

[31] Odutola A, Ota MOC, Antonio M, Ogundare EO, Saidu Y, Foster-Nyarko E (2017). Efficacy of a novel, protein-based pneumococcal vaccine against nasopharyngeal carriage of *Streptococcus pneumoniae* in infants: a phase 2, randomized, controlled, observer-blind study. Vaccine.

[32] Ojal J, Hammitt LL, Gaitho J, Scott JAG, Goldblatt D (2017). Pneumococcal conjugate vaccine induced IgG and nasopharyngeal carriage of pneumococCI hyporesponsiveness and immune correlates of protection for carriage. Vaccine.

[33] Nackers F, Cohuet S, le Polain de Waroux O, Langendorf C, Nyehangane D, Ndazima D (2017). Carriage prevalence and serotype distribution of *Streptococcus pneumoniae* prior to 10-valent pneumococcal vaccine introduction: a population-based cross-sectional study in South Western Uganda, 2014. Vaccine.

[34] Adetifa IMO, Adamu AL, Karani A, Waithaka M, Odeyemi KA, Okoromah CAN (2018). Nasopharyngeal pneumococcal carriage in Nigeria: a two-site, population-based survey. Sci Rep.

[35] Mohale T, Wolter N, Allam M, Ndlangisa K, Crowther-Gibson P, du Plessis M (2016). Genomic analysis of nontypeable pneumococci causing invasive pneumococcal disease in South Africa, 2003–2013. BMC Genomics.

[36] Iroh Tam PY, Thielen BK, Obaro SK, Brearley AM, Kaizer AM, Chu H (2017). Childhood pneumococcal disease in Africa-a systematic review and meta-analysis of incidence, serotype distribution, and antimicrobial susceptibility. Vaccine.

[37] Chaguza C, Cornick JE, Andam CP, Gladstone RA, Alaerts M, Musicha P (2017). Population genetic structure, antibiotic resistance, capsule switching and evolution of invasive pneumococci before conjugate vaccination in Malawi. Vaccine.

[38] Johnson HL, Deloria-Knoll M, Levine OS, Stoszek SK, Freimanis Hance L, Reithinger R (2010). Systematic evaluation of serotypes causing invasive pneumococcal disease among children under five: the pneumococcal global serotype project. PLoS Med.

[39] Birindwa AM, Kasereka JK, Gonzales-Siles L, Geravandi S, Mwilo M, Tudiakwile LK (2021). Bacteria and viruses in the upper respiratory tract of Congolese children with radiologically confirmed pneumonia. BMC Infect Dis.

[40] Adebanjo T, Lessa FC, Mucavele H, Moiane B, Chauque A, Pimenta F (2018). Pneumococcal carriage and serotype distribution among children with and without pneumonia in Mozambique, 2014–2016. PLoS ONE.

[41] Paton JC, Trappetti C (2019). *Streptococcus pneumoniae* capsular polysaccharide. Microbiol Spectr..

[42] Weinberger DM, Harboe ZB, Sanders EA, Ndiritu M, Klugman KP, Ruckinger S (2010). Association of serotype with risk of death due to pneumococcal pneumonia: a meta-analysis. Clin Infect Dis.

[43] Greenberg D, Givon-Lavi N, Newman N, Bar-Ziv J, Dagan R (2011). Nasopharyngeal carriage of individual *Streptococcus pneumoniae* serotypes during pediatric pneumonia as a means to estimate serotype disease potential. Pediatr Infect Dis J.

[44] Ominde M, Sande J, Ooko M, Bottomley C, Benamore R, Park K (2018). Reliability and validity of the World Health Organization reading standards for paediatric chest radiographs used in the field in an impact study of pneumococcal conjugate vaccine in Kilifi, Kenya. PLoS ONE.

[45] Lucero MG, Dulalia VE, Nillos LT, Williams G, Parreno RA, Nohynek H (2009). Pneumococcal conjugate vaccines for preventing vaccine-type invasive pneumococcal disease and X-ray defined pneumonia in children less than two years of age. Cochrane Database Syst Rev.

[46] Silaba M, Ooko M, Bottomley C, Sande J, Benamore R, Park K (2019). Effect of 10-valent pneumococcal conjugate vaccine on the incidence of radiologically-confirmed pneumonia and clinically-defined pneumonia in Kenyan children: an interrupted time-series analysis. Lancet Glob Health.

[47] Chappuy H, Keitel K, Gehri M, Tabin R, Robitaille L, Raymond F (2013). Nasopharyngeal carriage of individual *Streptococcus pneumoniae* serotypes during pediatric radiologically confirmed community acquired pneumonia following PCV7 introduction in Switzerland. BMC Infect Dis.

[48] Piralam B, Prosperi C, Thamthitiwat S, Bunthi C, Sawatwong P, Sangwichian O (2020). Pneumococcal colonization prevalence and density among Thai children with severe pneumonia and community controls. PLoS ONE.

[49] Sutcliffe CG, Shet A, Varghese R, Veeraraghavan B, Manoharan A, Wahl B (2019). Nasopharyngeal carriage of *Streptococcus pneumoniae* serotypes among children in India prior to the introduction of pneumococcal conjugate vaccines: a cross-sectional study. BMC Infect Dis.

[50] Sjostrom K, Spindler C, Ortqvist A, Kalin M, Sandgren A, Kuhlmann-Berenzon S (2006). Clonal and capsular types decide whether pneumococci will act as a primary or opportunistic pathogen. Clin Infect Dis.

[51] Fletcher MA, Schmitt HJ, Syrochkina M, Sylvester G (2014). Pneumococcal empyema and complicated pneumonias: global trends in incidence, prevalence, and serotype epidemiology. Eur J Clin Microbiol Infect Dis.

[52] Bozio CH, Abdul-Karim A, Abenyeri J, Abubakari B, Ofosu W, Zoya J (2018). Continued occurrence of serotype 1 pneumococcal meningitis in two regions located in the meningitis belt in Ghana five years after introduction of 13-valent pneumococcal conjugate vaccine. PLoS ONE.

[53] Birindwa AM, Emgard M, Norden R, Samuelsson E, Geravandi S, Gonzales-Siles L (2018). High rate of antibiotic resistance among pneumococci carried by healthy children in the eastern part of the Democratic Republic of the Congo. BMC Pediatr.

[54] Lindstrand A, Kalyango J, Alfven T, Darenberg J, Kadobera D, Bwanga F (2016). Pneumococcal carriage in children under five years in Uganda-will present pneumococcal conjugate vaccines be appropriate?. PLoS ONE.

[55] Emgard M, Msuya SE, Nyombi BM, Mosha D, Gonzales-Siles L, Norden R (2019). Carriage of penicillin-non-susceptible pneumococci among children in northern Tanzania in the 13-valent pneumococcal vaccine era. Int J Infect Dis.

[56] Satzke C, Turner P, Virolainen-Julkunen A, Adrian PV, Antonio M, Hare KM (2013). Standard method for detecting upper respiratory carriage of *Streptococcus pneumoniae*: updated recommendations from the World Health Organization Pneumococcal Carriage Working Group. Vaccine.

[57] Park IH, Geno KA, Sherwood LK, Nahm MH, Beall B (2014). Population-based analysis of invasive nontypeable pneumococci reveals that most have defective capsule synthesis genes. PLoS ONE.

[58] Weinberger DM, Warren JL, Dalby T, Shapiro ED, Valentiner-Branth P, Slotved HC (2019). Differences in the impact of pneumococcal serotype replacement in individuals with and without underlying medical conditions. Clin Infect Dis.

[59] Amin-Chowdhury Z, Collins S, Sheppard C, Litt D, Fry NK, Andrews N (2020). Characteristics of invasive pneumococcal disease caused by emerging serotypes after the introduction of the 13-valent pneumococcal conjugate vaccine in england: a prospective Observational Cohort Study, 2014–2018. Clin Infect Dis.

[60] Bergman K, Harnqvist T, Backhaus E, Trollfors B, Dahl MS, Kolberg H (2021). Invasive pneumococcal disease in persons with predisposing factors is dominated by non-vaccine serotypes in Southwest Sweden. BMC Infect Dis.

